# Heat shock factor 1 suppression induces spindle abnormalities and sensitizes cells to antimitotic drugs

**DOI:** 10.1186/s13008-021-00075-8

**Published:** 2021-12-18

**Authors:** Hsiao-Hui Kuo, Zhi-Rou Su, Jing-Yuan Chuang, Ling-Huei Yih

**Affiliations:** 1 Institute of Cellular and Organismic Biology, Academia Sinica, Taipei, 115 Taiwan; 2 Department of Medical Laboratory Science and Biotechnology, China Medical University, Taichung, Taiwan

**Keywords:** HSF1, Spindle assembly, Cell death

## Abstract

**Background:**

Heat shock factor 1 (HSF1) is the master regulator of the heat shock response and supports malignant cell transformation. Recent work has shown that HSF1 can access the promoters of heat shock proteins (HSPs) and allow HSP expression during mitosis. It also acts as a mitotic regulator, controlling chromosome segregation. In this study, we investigated whether the transactivation activity of HSF1 is required for the assembly of mitotic spindles.

**Results:**

Our results showed that phosphorylation of HSF1 at serine 326 (S326) and its transactivation activity were increased during mitosis. Inhibition of the transactivation activity of HSF1 by KRIBB11 or CCT251263 during mitosis significantly increased the proportion of mitotic cells with abnormal spindles. It also hampered the reassembly of spindle microtubules after nocodazole treatment and washout by impeding the formation of chromosomal microtubule asters. Depletion of HSF1 led to defects in mitotic spindle assembly, subsequently attenuating cell proliferation and anchorage-independent cell growth (AIG). These HSF1 depletion-induced effects could be rescued by ectopically expressing wild-type HSF1 or a constitutively active mutant (∆202-316, caHSF1) but not the S326A or dominant negative (∆361-529, dnHSF1) mutants. In addition, overexpression of HSP70 partially reduced HSF1 depletion-induced spindle abnormalities. These results indicate that HSF1 may support cell proliferation and AIG by maintaining spindle integrity through its transactivation activity. Furthermore, inhibition of HSF1 transactivation activity by KRIBB11 or CCT251236 can enhance diverse anti-mitosis drug-induced spindle defects and cell death.

**Conclusions:**

The increased transactivation activity of HSF1 during mitosis appears to be required for accurate assembly of mitotic spindles, thereby supporting cell viability and probably AIG. In addition, inhibition of the transactivation activity of HSF1 may enhance the mitotic errors and cell death induced by anti-mitosis drugs.

## Background

Heat shock factor-1 (HSF1) is the master transcriptional regulator of the cellular responses to heat and a wide variety of other stresses [1]. In response to these stresses, the inactive monomer HSF1 forms a trimer and is hyperphosphorylated to become transcriptionally active. The active HSF1 then binds to the heat shock elements (HSEs) of target genes [e.g., heat shock protein (HSP) 27, HSP70, and HSP90] to drive their expression [1]. The resulting rapid and robust induction of the heat shock response (HSR) involves cellular adaptations and protection mechanisms that can counteract many environmental stresses and pathophysiological conditions [2]. The conserved genome-wide transcriptional program coordinated by HSF1 not only restores the normal protein folding environment, but it also modulates signaling pathways and metabolism networks to enhance cell survival under various stresses [3]. In addition to its actions on HSPs, HSF1 also controls the transcription of numerous other genes, largely in a temperature-independent manner. Many of these non-HSP target genes of HSF1 have been implicated in various physiological processes [4].

HSF1 participates in several steps of carcinogenesis [5]. A broad spectrum of cancers exhibit high levels of nuclear and active HSF1, suggesting that HSF1 might exert oncogenic effects and act as a key facilitator of diverse cancers [6, 7]. In addition, HSF1 activation has been associated with poor responses to radiotherapy [8] and with chemotherapeutic drug resistance [9], indicating HSF1 is a pivotal determinant of therapeutic outcome. Notably, the functions of HSF1 that promote malignancy may extend beyond its well-known roles in protein quality control [10].

It has been demonstrated that HSF1 can access the HSP promoters and allow HSP expression during mitosis [11], implying that HSF1 has a transcriptional activation function during mitosis. It has also been shown that heat-stressed mitotic cells with high expression of HSP70 mRNA and protein are better protected against heat-induced mitotic errors [12]. On the other hand, PC3 cells made to overexpress a dominant-negative HSF1 are largely arrested in metaphase and more sensitive to colcemid, an inhibitor of tubulin polymerization, as compared to vector control cells [13]. Thus, HSF1 may help cancer cells overcome aberrant mitosis and its cellular consequences. In addition, HSF1 is known to be required for spindle pole body duplication in yeast [14]. In cancer cells, HSF1 is phosphorylated by polo-like kinase 1 (PLK1) at serine 216, causing it to sequester Cdc20. This sequestration keeps Cdc20 away from the anaphase-promoting complex/cyclosome degradation system, thereby interfering with mitosis exit and promoting chromosome missegregation in cancer cells [15, 16]. These studies suggest that the transactivation activity of HSF1 not only protects cells in the event of mitotic failure, but it also might directly regulate mitosis-related machineries. In this study, we explore whether the transactivation activity of HSF1 during mitosis is important for spindle assembly and for cell susceptibility to antimitotic drugs. Our results show that the transactivation activity of HSF1 is required for accurate spindle assembly and its inhibition can enhance the spindle defects and cell death induced by diverse anti-mitosis drugs.

## Results

### S326 phosphorylation and transactivation activity of HSF1 is increased during mitosis

**Fig. 1:**
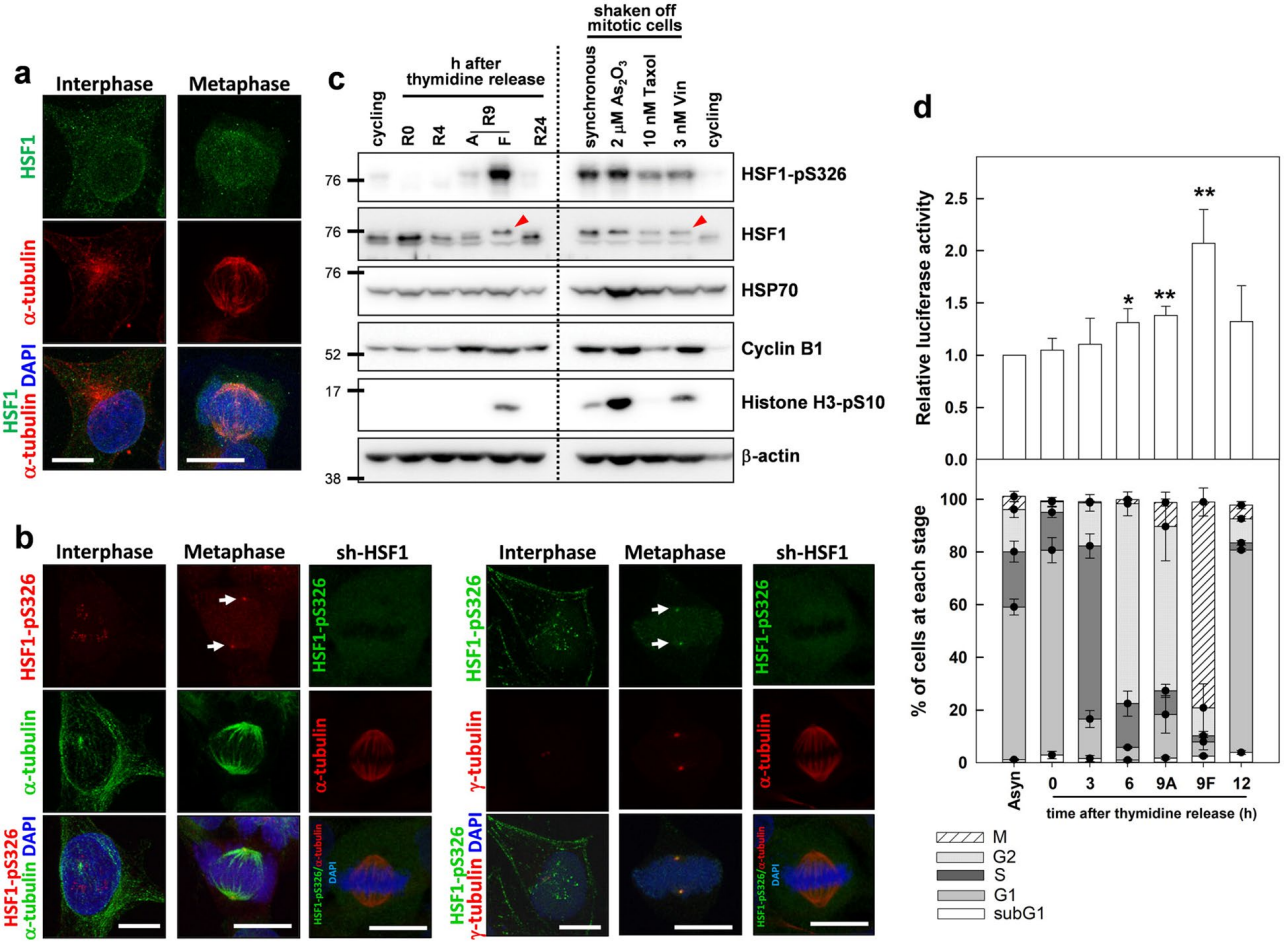
The distribution, expression and activity of HSF1. **a** Representative images show the cellular distributions of HSF1 in CGL2 cells at interphase (left panel) and metaphase (right panel). Logarithmically growing cells were fixed and stained with anti-HSF1 (green). The spindle microtubules were counterstained with anti-α-tubulin (red) and chromosomes with DAPI (blue). Scale bar, 10 μm. **b** Representative images show the cellular distributions of HSF1 phosphorylated at serine 326 (HSF1-pS326) in CGL2 cells at interphase and metaphase. Cells were immunostained with the two HSF1-pS326 antibodies shown in red (left panel, arrows) and in green (right panel, arrows) and respectively counterstained with anti-α-tubulin or anti-γ-tubulin. Arrows indicate the spots stained by HSF1-pS326 antibodies. sh-HSF1, cells depleted of HSF1 were used to test the antibody specificity and were immunostained with the corresponding HSF1-pS326 antibody (shown in green) and α-tubulin (shonw in red). **c** Levels of HSF1 and HSF1-pS326 at each cell cycle stage or in drug-arrested mitotic cells. CGL2 cells were enriched at each cell cycle stage by double-thymidine block and release as described in the section “Methods”, or cells were treated as indicated for 24 h. Cells were then collected by scraping the plate, or mitotic cells were collected by shaking off the plates. The collected samples were then used for immunoblot analysis. Red arrowhead indicates the shifted HSF1 band. **d** Transactivation activity of HSF1 at each cell cycle stage. CGL2-A1A-Luc cells were synchronized at the G1/S transition by double-thymidine block and then released from the block for the indicated times. The cells were then collected for analysis of luciferase activity assay or flow cytometry assay. 9A, the attached cells at 9 h after thymidine release. 9F, the floating cells at 9 h after thymidine release. Data shown are mean ± SD from three independent experiments. *p < 0.05 and **p < 0.01 by Student’s *t*-test as compared with the asynchronized cycling cells (Asyn)

HSF1 has been shown to colocalize with the kinetochore and spindle pole during mitosis [15, 16]. We thus examined whether HSF1 plays a role in spindle assembly. We first performed immunofluorescence staining to check the cellular distribution of HSF1 in CGL2 cells (a HeLa cell/normal human fibroblast hybrid) [17]. HSF1 did not show a distinct distribution in unstressed interphase CGL2 cells (Fig. 1a left panel), but faint staining was observed on the spindles of mitotic CGL2 cells (Fig. 1a right panel) using two different HSF1 antibodies (ADI-SPA-901 Enzo Life Sciences; sc-9144 Santa Cruz Technology). Similar results were found with both antibodies. In addition, Fig. 1b shows that HSF1 phosphorylated at serine 326 (HSF1-pS326) was prominently accumulated at the mitotic centrosome, according to immunostaining with two antibodies specific to HSF1-pS326 (Fig. 1b left, stained with Abcam ab76076; Fig. 1b right stained with Epitomics 2092-5. Cells depleted of HSF1 (Fig. 1b. sh-HSF1) was also stained with the HSF1-pS326 antibodies to check the specificity of the antibodies and the results showed that the spindle pole localization of phospho-HSF1 was diminished in the depleted cells, indicating that the antibodies are specific. Immunoblot analysis revealed a considerable elevation of HSF1-pS326 (Fig. 1c left) and a mobility shift of HSF1 (Fig. 1c left, arrowhead) only in shaken-off floating mitotic cells (F) of cultures released for 9 h from a double-thymidine block. These effects were not observed in the attached cells (A) or cells released for 0 or 4 h, confirming that the induction of HSF1 phosphorylation at S326 occurs during mitosis. Elevated expression of cyclin B1 and histone H3 phosphorylated at serine 10 (H3-pS10) confirmed that the cells were at the G2 and mitotic stages at 9 h after thymidine release (R9A and R9F). These findings are consistent with a previous report showing HSF1 had a shift in molecular weight during mitosis [11]. Correspondingly, the expression of HSP70 was also slightly increased in the floating mitotic cells. In addition, the levels of HSF1-pS326 and HSP70 were elevated in mitotic cells shaken off from cultures treated with anti-mitosis drugs (Fig. 1c, right). Since HSF1 phosphorylation at S326 is essential for its transactivation activity [18], our results imply that HSF1 might be transcriptionally active during mitosis. However, the amount of activated HSF1 trimers at the accessible region of the mitotic chromatin might be too low to be detected by immunofluorescence staining in our study.

To test whether the transactivation activity of HSF1 is indeed elevated at the mitotic stage, a stable luciferase HSF1 reporter CGL2 cell line containing firefly luciferase under the control of the HSPA1A promoter [19] was used to analyze HSF1 activity at each cell cycle stage. Figure 1d (bottom panel) shows the percentages of cells at each stage of the cell cycle in cultures released from a double-thymidine block for 0, 3, 6, 9, and 12 h. The upper panel shows that the attached G2 (9A) and floating mitotic cells (9F) enriched 9 h after release from a double-thymidine block exhibited a slightly but significantly higher level of luciferase activity than the cells at other cell cycle stages. This result implies that the transactivation activity of HSF1 was increased during G2 and mitotic stages, which is in agreement with our observations that the levels of HSF1-pS326 and HSP70 were considerably increased in mitotic cells (Fig. 1c). Together, these results show that HSF1 transactivation activity is increased during mitosis.

### Inhibition of HSF1 transactivation activity during mitosis induces spindle defects and interferes with the reassembly of mitotic spindles

**Fig. 2:**
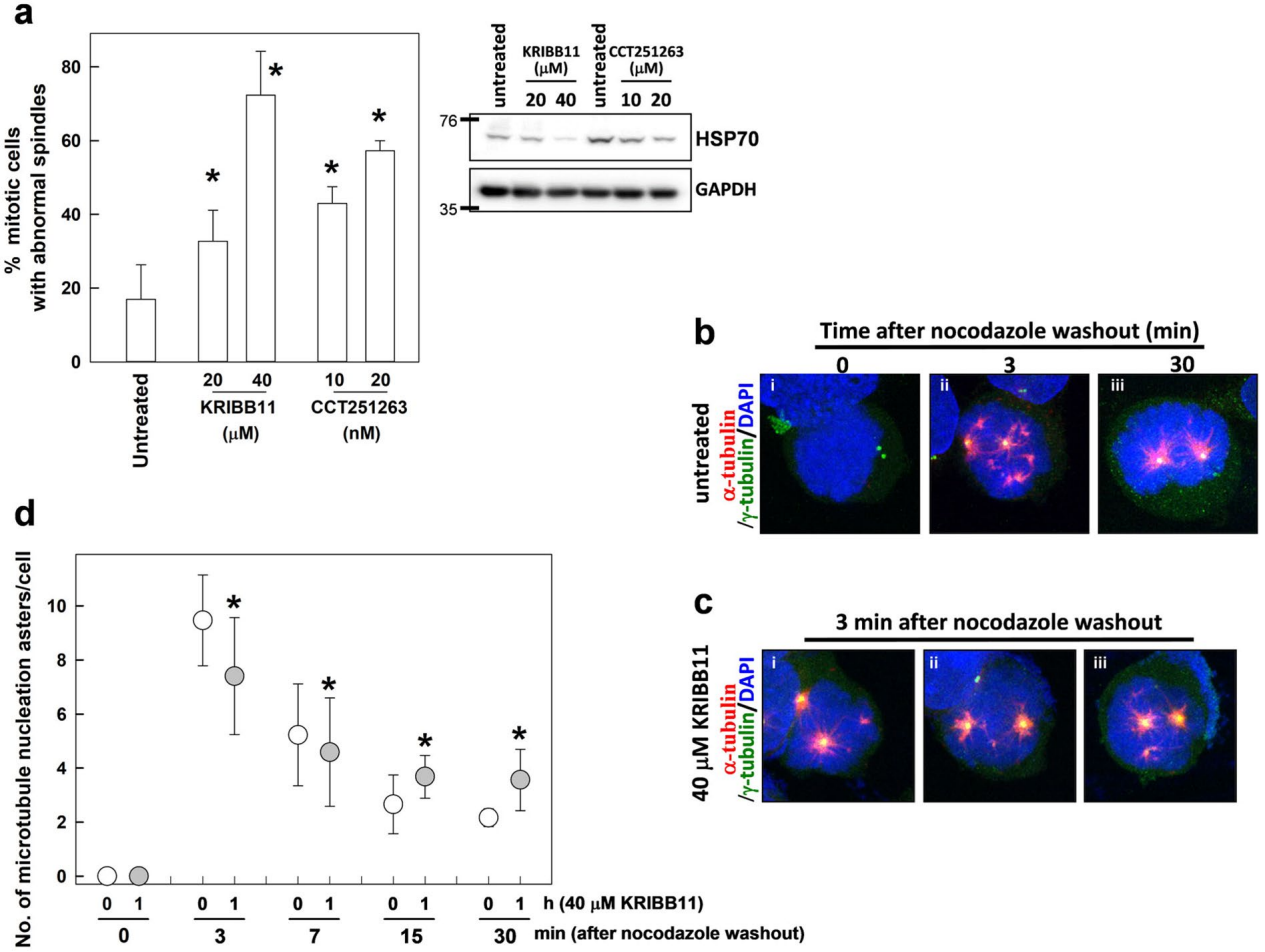
Inhibition of HSF1 transactivation activity during mitosis induces spindle abnormalities and alters the reassembly of mitotic spindles. **a** Treatment of KRIBB11 and CCT251263 at mitotic stage induced defects in mitotic spindles. CGL2 cells were treated with indicated concentrations of KRIBB11 or CCT251263 for 1 h and immediately subjected to analysis of mitotic spindles by immunofluorescence staining. The results (mean ± SD) were derived from at least 500 mitotic cells from three independent experiments. *p < 0.01 by Student’s *t*-test as compared with the untreated control. After treatment, the mitotic cells were also shaken off the plates and collected for immunoblot analysis of HSP70 (right panal). **b** Representative images of the mitotic spindle after nocodazole treatment (3 μM for 0.5 h) and washout for 0 (**i**), 3 (**ii**), and 30 min (**iii**). **c** CGL2 cells were treated with 40 μM KRIBB11 for 1 h; during the last 0.5 h of KRIBB11 treatment, nocodazole was added into culture medium at 3 μM. Nocodazole was then completely washed out, and the cells were incubated in drug-free medium for 3 min before being immediately fixed and immunostained for α-tubulin (red) and γ-tubulin (green). The chromosomes were counterstained with DAPI (blue). **d** The numbers of microtubule asters in cells. Results are the median ± 25th percentiles of at least 250 mitotic cells for each condition, as determined from three experiments. *p < 0.05 by Dunnett test as compare every median with a KRIBB11-untreated control median using the GraphPad Prism 9.1

We next examined whether the transactivation activity of HSF1 is required at the mitotic stage for spindle assembly. To suppress HSF1 transactivation activity at the mitotic stage and examine the consequent effects on spindle assembly, CGL2 cells were treated for 1 h with KRIBB11 or CCT251236, both of which are inhibitors of HSF1 transactivation [20, 21]. Mitotic cells were then immediately analyzed for the assembly of mitotic spindles. The results showed that treatment with KRIBB11 or CCT251236 for 1 h markedly induced defects in mitotic spindles (Fig. 2a, left), indicating that HSF1 transactivation activity is required at mitosis for accurate assembly of spindles. The mitotic cells were also shook off the plates and collected for immunoblot analysis of HSP70 expression. The results showed that HSP70 expression was considerably decreased in these cells (Fig. 2a, right), indicating HSF1 activity was decreased by these inhibitors.

A nocodazole washout experiment [22] was then performed to assess whether HSF1 transactivation activity is involved in the regulation of microtubule polymerization for spindle assembly. The mitotic spindle was completely disassembled after a 0.5-h treatment of 3 μM nocodazole, leaving two centrosomes (visualized by staining of γ-tubulin, Fig. 2b—i, green) in the mitotic cell, as revealed by immunofluorescence staining of the mitotic spindle with antibodies against α- and γ-tubulin. After a thorough washout of nocodazole and re-incubation in drug-free medium for 3 min, the microtubule fibers (visualized by staining of α-tubulin, red) gradually formed large asters around the two centrosomes and many tiny asters within chromosomes (Fig. 2b—ii). After 30 min, the chromosome-associated microtubule asters disappeared, likely having merged into the two centrosomal asters, and a bipolar mitotic spindle reformed (Fig. 2b—iii). This sequence of events is consistent with a previous report [23]. However, in the presence of KRIBB11, the number of chromosome-associated microtubule asters was significantly lower than that in the non-KRIBB11-treated cells at 3 min after nocodazole washout (Fig. 2c and d). In addition, reassembly of the bipolar mitotic spindle after nocodazole washout in KRIBB11-treated mitotic cells was delayed, as the number of microtubule asters was significantly higher in KRIBB11-treated cells than in the non-KRIBB11-treated cells at 15 and 30 min after nocodazole washout (Fig. 2d). Since cells rely on chromatin-driven microtubule assembly to form a bipolar spindle [23], these results indicate that the transactivation activity of HSF1 is required for assembly of a bipolar mitotic spindle.

### Depletion of HSF1 induces spindle abnormalities, alters mitosis progression, and impedes cell proliferation

**Fig. 3:**
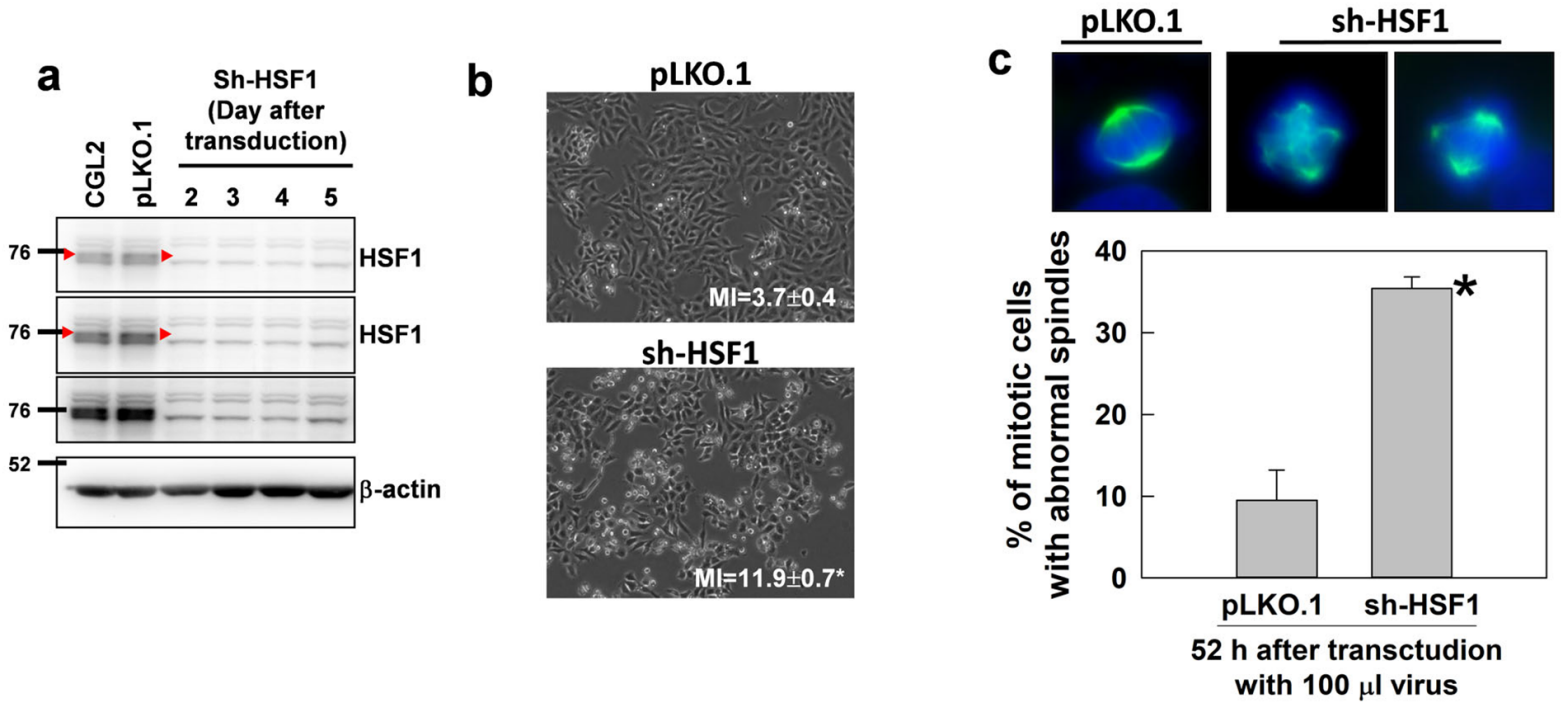
Depletion of HSF1 induces spindle abnormalities. **a** Depletion efficiency of HSF1. CGL2 cells were transduced with virions containing no shRNA (pLKO.1) or HSF1-specific shRNA (sh-HSF1). Beginning at 50 h post-transduction, the cells were collected daily for analysis of HSF1 depletion efficiency by immunoblotting. Red arrowheads indicate the positions of HSF1 bands. **b** Representative images of CGL2 cells 52 h after transduction with virions containing no shRNA (pLKO.1) or HSF1-specific shRNA (sh-HSF1). Mitotic indices (MIs) were determined by counting DAPI-stained interphase and mitotic cells at 40× magnification using a fluorescence microscope. At least 1000 cells were counted. Data presented are the mean ± SD from three independent experiments. **c** HSF1 depletion induced spindle abnormalities. Cells were transduced as described in **b** and then subjected to mitotic spindle analysis by immunofluorescence staining. Representative images show mitotic spindles from CGL2 cells transduced with virions containing pLKO.1 control or HSF1 shRNA. The results (mean ± SD) were determined from at least 300 mitotic cells from three independent experiments. *p < 0.01 by Student’s *t*-test as compared with cells transduced with pLKO.1 control virion

We next tested whether HSF1 regulates spindle assembly and thus affects cell growth by transducing CGL2 cells with virions containing HSF1-specific shRNA. Figure 3a shows that the expression of HSF1 was efficiently reduced at 2–5 days after transduction of CGL2 cells with virion containing shRNA targeting HSF1 (sh-HSF1). Figure 3b shows the morphology of CGL2 cells at 52 h after transduction with the control virion (pLKO.1, upper) and virion containing sh-HSF1 (lower). The portion of rounded-up cells and the mitotic index (MI) were significantly increased in cultures depleted of HSF1 (Fig. 3b). In addition, immunofluorescence staining of mitotic spindles with α-tubulin antibody revealed that 52 h after transduction of cells with HSF1-specific shRNA, considerable abnormalities in mitotic spindles could be detected (Fig. 3c). An increase in MI indicates the accumulation or arrest of mitotic cells in cultures, and it is widely recognized that the length of mitosis correlates with the occurrence of mitotic errors in the cells [11]. Our results thus may suggest that cells with decreased HSF1 levels are subject to more mitotic errors.

**Fig. 4:**
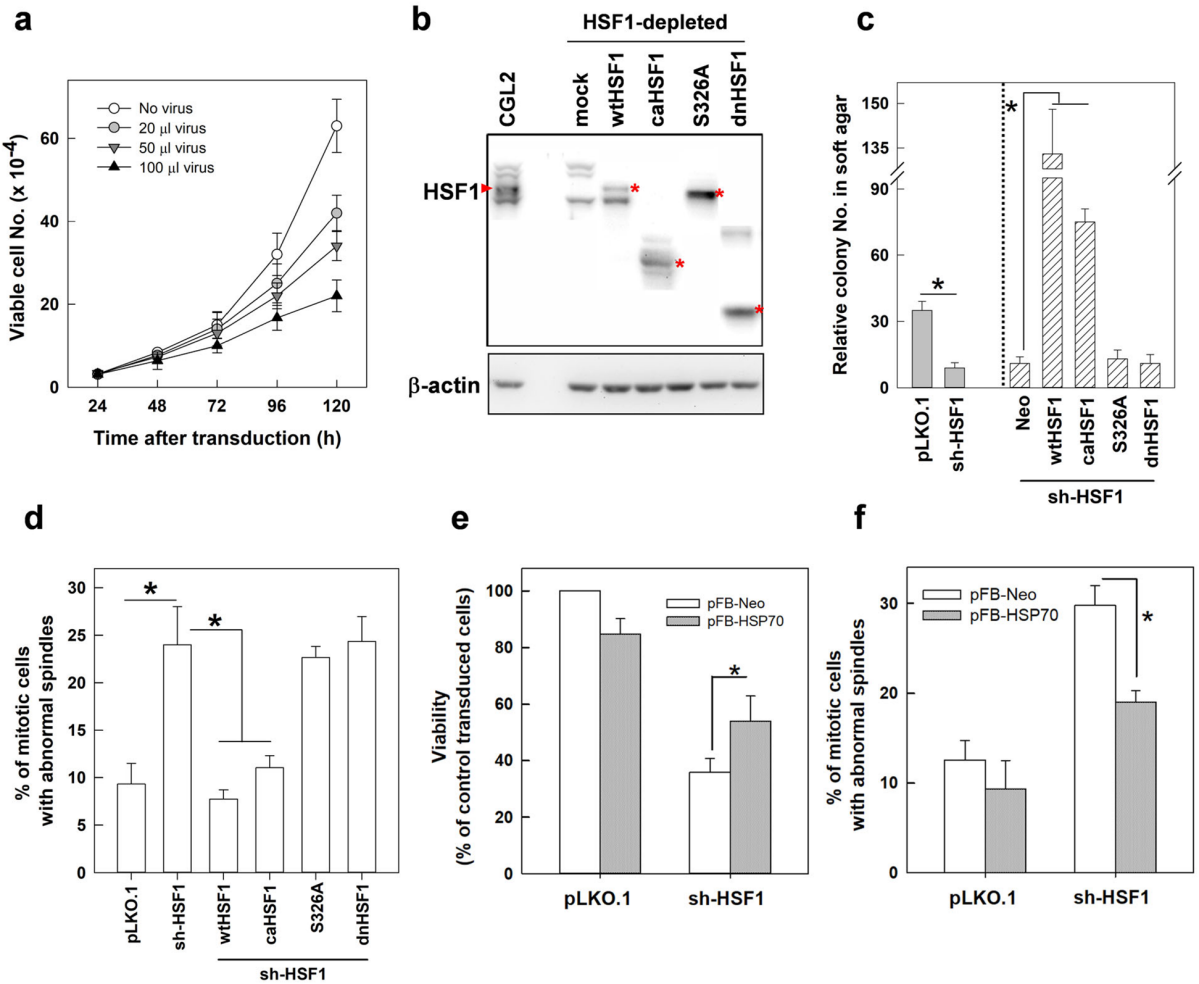
Transactivation activity of HSF1 is required for cell proliferation, AIG, and assembly of mitotic spindles. **a** Depletion of HSF1 attenuated cell proliferation. Cells were transduced with various volumes of virions containing HSF1 shRNAs or not, and then cell proliferation was analyzed daily by the trypan blue exclusion assay. **b** Immunoblot analysis of the levels of endogenous HSF1 in CGL2 cells and the FLAG-tagged wild-type and mutant HSF1 in HSF1-depleted cells. Red asterisk indicates the position of each HSF1 expressed in the cells. **c** and **d** Depletion of HSF1 suppressed AIG and induced spindle abnormalities. Overexpression of HSF1 isoforms lacking transactivation activity could not rescue HSF1 depletion-induced AIG suppression and spindle defects. HSF1-depleted cells were transduced with virions containing cDNAs of FALG-tagged wild-type (wt) HSF1 or mutant HSF1 for 50 h and then collected for immunoblotting analysis (**b**), AIG (**c**), or spindle abnormalities (**d**). **e** and **f** Overexpression of HSP70 reduced the cell death and rescued the spindle defects induced by HSF1 depletion. The means ± SD from at least three independent experiments are shown. *p < 0.01 by Student’s *t* test

Subsequent to inducing spindle defects and altering mitosis progression at 52 h after transduction of HSF1-specific shRNA, the proliferation of HSF1-depleted cells was impaired, as indicated by the significant decrease of viable cells compared to those with no virus transduction (Fig. 4a). In addition, anchorage-independent cell growth (AIG) was also considerably impaired in cells depleted of HSF1 when compared to those transduced with empty control virion (Fig. 4c left, pLKO.1 vs sh-HSF1). These results suggest that depletion of HSF1 might first induce defects in mitotic spindles, then it may impair mitosis progression and cell proliferation, consequently obstructing AIG.

Whether the transactivation activity of HSF1 is required to maintain functional bipolar spindles and AIG was then tested in cells depleted of HSF1. CGL2 cells depleted of HSF1 were made to ectopically overexpress wild-type HSF1 (wtHSF1), constitutively active HSF1 (∆202-316, caHSF1), S326A substituted HSF1, or dominant negative HSF1 (∆361-529, dnHSF1), as previously described [19]. The effects of each mutant HSF1 on HSP70 expression have been previously reported [19]. The respective depletion and overexpression efficiencies were confirmed by immunoblotting (Fig. 4b). The effects of wild-type and mutant HSF1 isoforms on the induction of abnormal mitotic spindles and AIG in cells depleted of HSF1 were then analyzed. Figure 4d shows that expression of wtHSF1 or caHSF1 but not S326A or dnHSF1significantly reduced the induction of abnormal mitotic spindles by HSF1 depletion. The reduction of AIG by HSF1 depletion was also recovered and further enhanced by overexpressing wtHSF1 or caHSF1 but not S326A or dnHSF1 (Fig. 4c right). The inducible heat shock protein 70 (HSP70 encoded by *HSPA1A* gene) was also ectopically overexpressed in CGL2 cells depleted of HSF1. The results showed that HSF1 depletion-induced cell death (Fig. 4e, left) and spindle abnormalities (Fig. 4e, right) could be partially rescued by overexpression of HSPA1A. Therefore, we conclude that the transactivation activity of HSF1 is required for bipolar spindle assembly and AIG and that HSF1 may regulate the assembly of mitotic spindles partially through the expression of HSP70.

### Inhibition of HSF1 transactivation activity sensitizes cells to anti-mitosis drugs

**Fig. 5:**
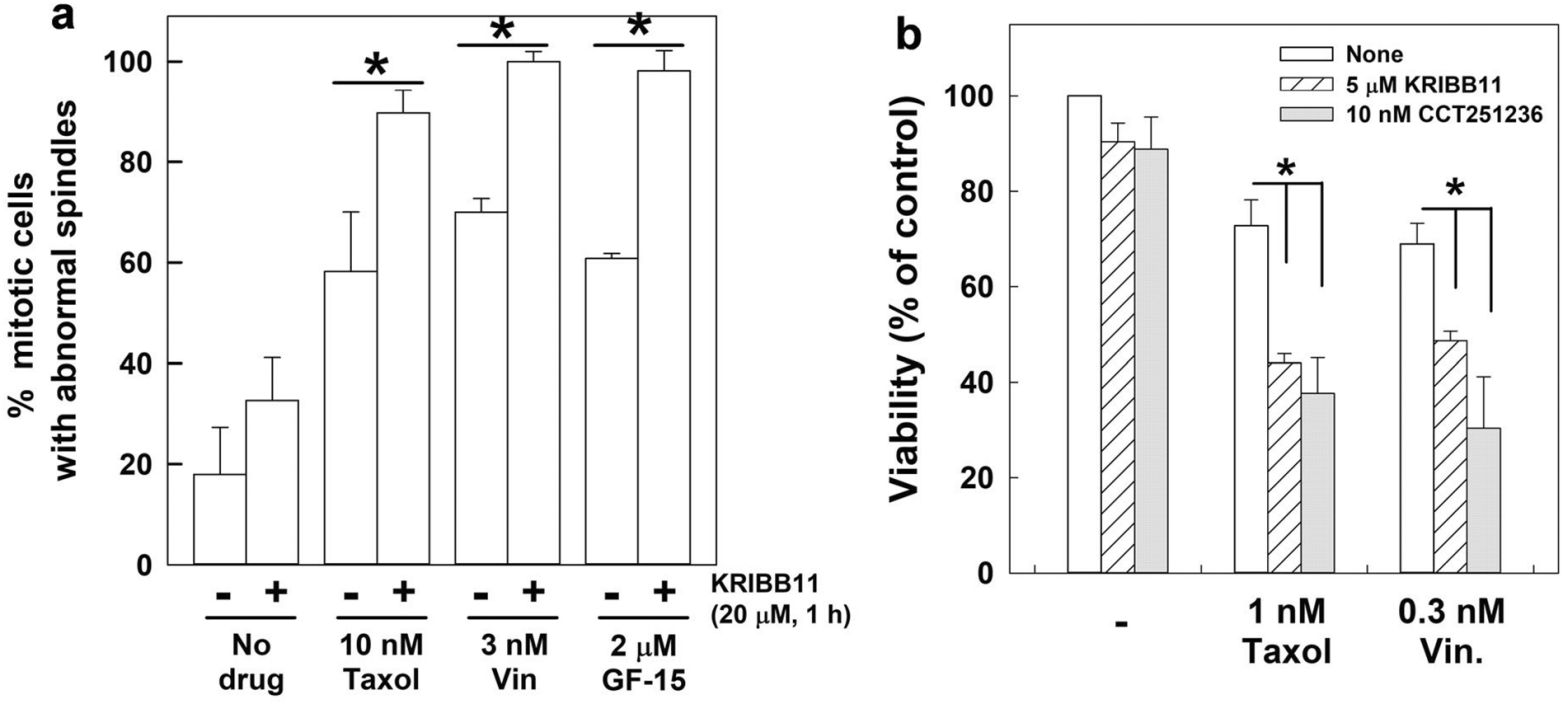
KRIBB11 enhances anti-mitosis drug-induced spindle defects and cell death in CGL2 cells. **a** Inhibition of HSF1 with KRIBB11 during mitosis enhanced anti-mitosis drug-induced spindle abnormalities. **b** Inhibition of HSF1 with KRIBB11 or CCT251236 enhanced cell death induced by anti-mitosis drugs. CGL2 cells were incubated in media containing KRIBB11 or CCT251236 alone or in combination with an anti-mitosis drug, i.e., Taxol, vinblastine (Vin), or GF-15, at the indicated concentrations for 1 h (**a**) or 72 h (**b**). Spindle abnormalities and cell viability were measured at the indicated times. The means ± SD from at least three independent experiments are shown. *p < 0.05 by Student’s *t* test

**Fig. 6:**
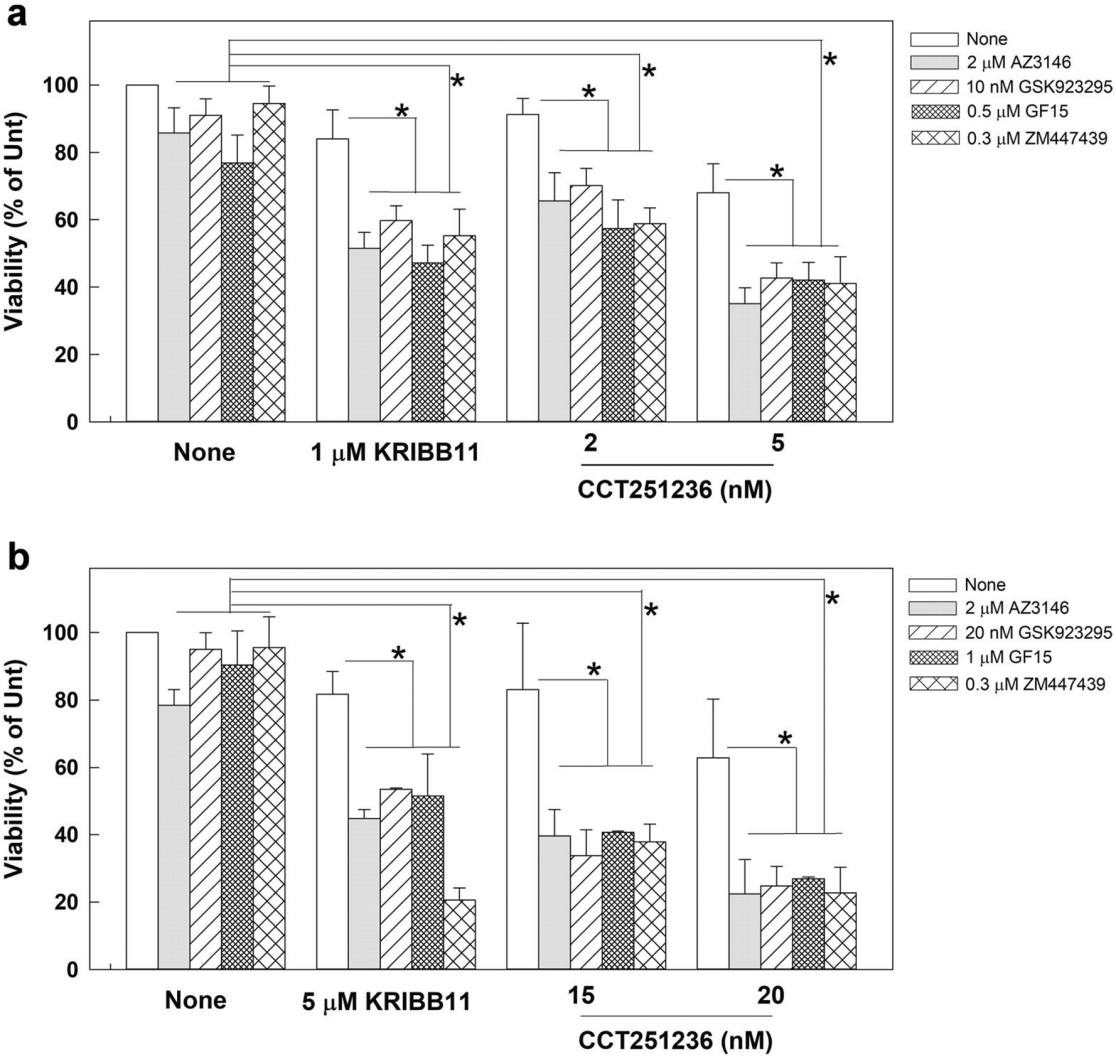
Inhibition of HSF1 transactivation activity enhances cell death-induced by diverse anti-mitosis drugs. CGL2 (**a**) and MDA-MB-231 (**b**) cells were incubated in media containing HSF1 inhibitor, KRIBB11 or CCT251236, alone or in combination with anti-mitosis drugs, AZ3146, GSK923295, GF15, or ZM447439. Drugs were treated at the indicated concentrations for 72 h, after which cell viability was measured. The means ± SD from at least three independent experiments are shown. *p < 0.05 by Student’s *t* test

Since the level of HSF1-pS326 was elevated in the mitotic cells collected from anti-mitosis drug-treated cultures (Fig. 1c), we next assessed whether the transactivation activity of HSF1 could modulate the cell response to anti-mitosis drugs by treating cells with KRIBB11 and CCT251236 and monitoring spindle defects and cell death. The results showed that cotreatment of CGL2 cells with KRIBB11 and Taxol, vinblastine, or GF15 (a potent inhibitor of centrosome clustering) for 1 h could significantly enhance the spindle abnormalities compared to the corresponding treatments with each anti-mitosis drug alone (Fig. 5a). In addition, the 72 h-viability assay revealed that KRIBB11 or CCT251236 also significantly increased the level of cell death induced by Taxol or vinblastine (Fig. 5b). A series of anti-mitotic drugs were also examined, including AZ3146 (a potent and specific inhibitor of monopolar spindle 1 kinase, MPS1), GSK923295 (a potent and selective inhibitor of the mitotic kinesin centromere-associated protein-E, CENP-E), GF15 (a centrosomes clustering inhibitor), or ZM447439 (a potent and selective inhibitor of aurora B kinase, AURKB). Notably, each anti-mitotic drug at the concentration applied only caused minor cytotoxicity in the culture. We then treated CGL2 (Fig. 6a) or MDA-MB-231 breast cancer cells (Fig. 6b) with each anti-mitotic drug plus the indicated HSF1 inhibitor and found that the combined treatment significantly reduced the cell viability compared to anti-mitosis drug alone or HSF1 inhibitor alone (Fig. 6). In addition, according to the combination index (CI) theory of Chou-Talalay [24], the CI of our combined treatments are smaller than 1, suggesting that HSF1 inhibitor could enhances the cytotoxicity of each anti-mitotic drug. These results suggest that HSF1 transactivation activity might not only be required for accurate assembly of mitotic spindles and successful mitosis progression, but it may also protect mitotic cells from diverse anti-mitosis drugs. Thus, inhibition of HSF1 might concomitantly induce spindle defects and inhibit its cytoprotective function, sensitizing cells to diverse anti-mitosis drugs.

## Discussion

In this study, we showed that the S326 phosphorylation and transactivation activity of HSF1 was elevated during mitosis (Fig. 1c) and that the phospho-HSF1 was localized at the spindle pole at metaphase (Fig. 1b). Then, using HSF1 transactivation inhibitors or cells depleted of HSF1 with added-back constitutively active or dominant negative mutants, we provided evidence that the transactivation activity of HSF1 at the mitotic stage is required for spindle assembly.

Phosphorylation of HSF1 at S326 plays a critical role in its transactivation [18], and several kinases, including mTOR [25], MEK [26] and p38 MAPK [27], have been shown to perform this phosphorylation. These kinases are known to be activated during mitosis [28, 29]; however, whether they might induce the mitotic activation of HSF1 requires further investigation. Autophosphorylation of mTOR at S2481 correlates with mTOR catalytic activity [30], and active mTOR accumulates at mitotic centrosomes and spindles, potentially acting as a regulator of mitotic spindle assembly [31]. A molecular and functional link between HSF1, mTOR, and spindle assembly during mitosis might therefore be expected and warrants further study. Moreover, PLK1 is essential for mitosis progression due to its effects on maturation of mitotic centrosomes, assembly of mitotic spindle, and cytokinesis [32]. PLK1 has been shown to phosphorylate HSF1 at S419 upon heat stress to induce HSF1 nuclear translocation [33]. Furthermore, PLK1 phosphorylates HSF1 at S216 during mitosis [15, 16]. This S216-phosphorylated HSF1 localizes to the spindle pole and binds Cdc20 in early mitosis, sequestering Cdc20 from the anaphase-promoting complex and affecting mitosis progression. However, whether this PLK1-phosphorylated, spindle pole-localized HSF1 plays a role in spindle assembly has not been shown. Regardless of the specific mechanisms, our results demonstrate that inhibition of the transactivation activity of HSF1 during mitosis can induce spindle abnormalities (Fig. 2), suggesting that HSF1 might remain transcriptionally active during mitosis to support bipolar spindle assembly.

It has been demonstrated that the condensed chromatin of mitotic cells is still accessible to HSF1 and RNA polymerase II, allowing downstream expression of HSPs [11]. Our results showed that the expression of HSP70 was elevated in the mitotic cells collected from thymidine block-and-release cultures and those arrested by treatment of diverse anti-mitosis drugs. The expression levels of several other HSPs and chaperones were also reported to be increased in nocodazole- or colcemid-arrested mitotic cells [34]. HSP70 regulates the function of mitotic centrosomes [35, 36], and HSP90 activity is required for sister chromatid cohesion and precise chromosome segregation during mitosis [37]. It has also been reported that several regulatory proteins involved in controlling mitosis progression are stabilized by molecular chaperones. Cyclin-dependent kinase 1 (CDK1) is the engine kinase for mitosis entry, as it drives a large number of interconnected signaling cascades [38]; this protein has been shown to form a complex with HSP90 to mediate signal transduction during mitosis and cytokinesis [39]. In addition, tubulin has been shown to complex with HSP70 and t complex polypeptide-1 in order to enable tubulin folding during the assembly and disassembly of the mitotic apparatus [40]. These studies indicate that several HSPs and chaperones may participate in and be required for spindle assembly and mitosis progression. Thus, HSF1 may act via its function as a master regulator of HSPs and chaperones to control the mitotic apparatus and mitosis progression. Alternatively, HSF1 has been demonstrated to bind the promoter of Forkhead box M1 (FoxM1) transcription factor to stimulate its expression [41]. Since FoxM1 is required for the transcription of genes involved in the G2/M transition and construction of the mitotic apparatus (targets include cyclin B1, Cdc20, CDK1 and PLK1, among others) [42], HSF1 might also control spindle assembly and mitosis progression by modulating the expression of FoxM1. Therefore, HSF1 could potentially control spindle assembly and mitosis progression through both direct and indirect mechanisms.

Our results also show that inhibition of HSF1 transactivation activity or depletion of HSF1 first induces defects in mitotic spindle assembly and subsequently inhibits cell proliferation and AIG. Dysregulation of signaling cascades involving oncogenes and tumor suppressor genes often leads to centrosome abnormalities and results in defects in mitotic spindles [43]. The subsequent chromosome mis-segregation and aneuploidy has been shown to induce mitotic catastrophe that eliminates mitosis-incompetent cells [44]; otherwise, cell proliferation may be compromised by inducing proteotoxicity in the aneuploid daughter cells [45]. As such, several reports have demonstrated that increased chaperone or HSF1expression rescues not only the protein folding defects in human aneuploid cells, but it also rescues proliferation defects [46] and protects cells from mitotic apoptosis [47–49]. In addition, expression of a dominant-negative HSF1 construct reduced the aneuploid population in a prostate cancer cell line [13]. Since aneuploid cells mainly arise due to mitotic failures, this finding indicates that inhibition of HSF1 activity may hamper the progression and survival of aberrant mitotic cells. Together, these studies suggest that transformed cells might depend on HSF1 signaling to cope with mitotic failures and maintain cell division, survival and proliferation. Thus, in addition to regulating spindle assembly, transcriptionally active HSF1 plays a role in cytoprotection during mitosis and aneuploidy-accompanying proteotoxicity; hence, HSF1 may facilitate AIG and tumorigenesis.

We also found that inhibition of HSF1 transactivation activity by KRIBB11 or CCT251236 could enhance the spindle defects and cell death induced by diverse anti-mitosis drugs. The anti-mitosis drugs we tested target different proteins, including tubulin, MPS1, CENP-E and AURKB, and inhibit centrosome clustering. The results indicate that HSF1 can generally protect from the diverse types of mitotic damage induced by these drugs. It was demonstrated that HSF1 transcriptionally activates the expression of multidrug-resistance 1 gene [50], indicating that HSF1 may protect cells by facilitating drug exclusion. In addition to its regulation of HSPs, HSF1 also controls the transcription of genes encoding other proteins, which are involved in various physiological processes [4]. Thus, HSF1 might participate in numerous signaling pathways during mitosis, including spindle assembly and cytoprotection, to sustain the survival of cells experiencing mitotic failures. Targeting the mitotic machinery with anti-mitosis drugs is routinely used as a first-line treatment for many cancers. However, patient responses are often unpredictable, and drug resistance frequently limits the effectiveness of these drugs. Therefore, our result showing that HSF1 suppression can sensitize cancer cells to anti-mitosis drugs has the potential to inspire improved clinical treatments.

## Conclusions

In summary, our results reveal that the transactivation activity of HSF1 during mitosis is required for spindle assembly. Inhibition of the transactivation activity of HSF1 can impair spindle assembly and mitosis progression, thus reducing cell viability and AIG. Additionally, inhibition of the transactivation activity of HSF1 also prevents its cytoprotective function, and cells with diminished HSF1 activity show increased levels of spindle defects and death upon treatment with diverse anti-mitosis drugs. Thus, inhibition of HSF1 might concomitantly induce spindle defects and inhibit its cytoprotective function, sensitizing cells to diverse anti-mitosis drugs.

## Methods

### Cell culture and chemicals

CGL2 cells were kindly provided by Dr. E. J. Stanbridge (University of California-Irvine). CGL2 and MDA-MB-231 cells were cultured as described [19, 51]. A stable HSF1 luciferase reporter cell line (CGL2-A1A-Luc) was established by G418 selection of CGL2 cells transfected with pGL4-A1A-1.3K plasmid [19], a luciferase reporter plasmid containing the heat shock element (HSE) from the promoter of the HSPA1A gene. Cell cycle synchronization at G1 was achieved by double-thymidine block [52]. Cells were then allowed to progress after switching to thymidine-free medium; the cell cycle progression monitored at 2–4 h intervals using a DNA flow cytometer. The cell viability assay was carried out as described [19].

AZ3146 (Cayman, Ann Arbor, MI, USA), CCT251236 (Cayman), KRIBB11 (Merck Calbiochem, MA, USA), GF15 (Merck Calbiochem), GSK923295 (Cayman), taxol (Merck Calbiochem), vinblastine (Sigma, St. Louis, MO, USA), and ZM447439 (Cayman) were prepared in DMSO and freshly diluted in culture medium before use.

### Expression vectors

The retroviral plasmids encoding either wild-type HSF1, constitutively active HSF1 (∆202-316, caHSF1), S326A HSF1, or dominant-negative HSF1 (∆361-529, dnHSF1) were prepared as described [19]. All sequences of wild-type and mutant HSF1 cDNAs were confirmed and verified by DNA sequencing. Cells were transduced or transfected with the plasmids as previously described [19].

### Depletion of cellular HSF1

Depletion of HSF1 was achieved by transducing CGL2 cells with VSV-G-pseudotyped lentivirus-based short hairpin RNA (shRNA) as previously described [19]. CGL2 cells were transduced with pLKO.1- or shRNA-containing virions in growth medium supplemented with 10 μg/ml polybrene. At 24 h post-transduction, 2 μg/ml puromycin was added to culture medium to select for stable clones (CGL2-pLKO.1 and CGL2-shHSF1). Alternatively, cells were subjected to analysis after transduction for at least two days.

### Analysis of cell cycle progression and mitotic index

Cell cycle progression was monitored using DNA flow cytometry as previously described [52]. The percentage of cells in mitosis was analyzed by direct microscopic observation of DAPI-stained cells or by flow cytometry analysis of phospho-histone H3 (mitosis marker)-positive cells.

### Immunoblotting

Cell lysis and immunoblotting were carried out as described [35]. Specific proteins were detected using antibodies against cyclin B1 (Santa Cruz Biotechnology, Inc., Dallas, Texas), histone H3 phosphorylated at S10 (Cell Signaling Technology, Danvers, MA), HSF1 (Enzo Life Sciences, Lausen, Switzerland), HSF1 phosphorylated at S326 (ab76076, Abcam, Cambridge, MA), and HSP70 (GeneTex, Hsinchu, Taiwan). β-Actin and GAPDH was detected with anti-β-actin (Chemicon, Temecula, CA) and anti-GAPDH (Genetex), respectively, for use as loading controls.

### Immunofluorescence staining

Cells seeded on glass coverslips were incubated for 24 h at 37 °C, washed twice with PBS, fixed in PTEMF (20 mM PIPES, 4% paraformaldehyde, 0.2% Triton-X, 10 mM EGTA and 1 mM MgCl_2_) for 15 min, and then immunostained as described [35]. The primary antibodies used include anti-α-tubulin (T5168, Sigma, Saint Louis, MO or GTX112141, GeneTex), anti-γ-tubulin (T6557 or T3559, Sigma), anti-HSF1 (ADI-SPA-901 Enzo Life Sciences, Lausen, Switzerland or SC-9144, Santa Cruz Technology), antibodies specific to HSF1 phosphorylated at S326 (HSP1-pS326, ab76076, Abcam or 2092-5, Epitomics, Inc., Burlingame, CA), anti-HSF1-pS303/307 (2108-5, Epitomics, CA). Alexa-Fluor 488- or 633-conjugated goat anti-mouse or anti-rabbit IgG were purchased from Invitrogen (Carlsbad, CA, USA). All primary antibodies were diluted in 1:200 in phosphate-buffered saline containing 0.1% tween 20. The nuclei were counterstained with 0.1 μg/ml of 4,6-diamino-2-phenyl-indole (DAPI, Sigma). Samples were mounted in Fluoromount-G from SouthernBiotech (Birmingham, AL, USA) and examined under a confocal microscope (Leica TCS-SP5, Mannheim, Germany).

### Nocodazole washout assay

The nocodazole washout assay was used to assess the effect of HSF1 on assembly of mitotic spindles. The mitotic spindles in control or KRIBB11-treated mitotic cells were dissolved by incubating the cells with 3 μM nocodazole for 0.5 h. Afterward, nocodazole was rapidly rinsed off, and the cells were carefully transferred to 37 °C drug-free medium. At various time points (0, 3, 7, 15, 30 min), cells were fixed with PTEMF for 15 min and stained with anti-α-tubulin and anti-γ-tubulin antibodies; the tubulins were visualized by staining with Alexa488- or Alexa633-conjugated secondary antibodies. Re-assembly of the mitotic spindle was detected using a fluorescence microscope (Zeiss Axioplan 2 Imaging MOT, Oberkochen, Germany).

## Data Availability

The data that support the findings of this study are available from the corresponding author upon reasonable request.

## Abbreviations

AIG: Anchorage-independent cell growth
AURKB: Aurora B kinase
CDK1: Cyclin-dependent kinase 1
CENP-E: Mitotic kinesin centromere-associated protein-E
FoxM1: Forkhead box M1
HSE: Heat shock element
HSF1: Heat shock factor 1
HSP: Heat shock protein
HSR: Heat shock response
MI: Mitotic index
MPS1: Monopolar spindle 1 kinase
PLK1: Polo-like kinase 1
